# Hemithyroidectomy, does the indication influence the outcome?

**DOI:** 10.1007/s00423-023-03168-w

**Published:** 2023-12-07

**Authors:** Ervin Beka, Hanan Hanna, Pia Olofsson, Oliver Gimm

**Affiliations:** 1 Department of Surgery and Department of Biomedical and Clinical Sciences, Linköping University, Linköping, Sweden; 2 Department of Hand Surgery, Plastic Surgery and Burns, Linköping University, Linköping, Sweden

**Keywords:** Thyroid gland, Hemithyroidectomy, Register study, Complications

## Abstract

**Purpose:**

Hemithyroidectomies are mainly performed for two indications, either therapeutically to relieve compression symptoms or diagnostically for suspicious nodule(s). In case of the latter, one could consider the approach to be rather extensive since the majority of patients have no symptoms and will have benign disease. The aim of this study is to investigate the complication rates of diagnostic hemithyroidectomy and to compare it with the complication rates of compressive symptoms hemithyroidectomy.

**Methods:**

Data from patients who had undergone hemithyroidectomy either for compression symptoms or for excluding malignancy were extracted from a well-established Scandinavian quality register (SQRTPA). The following complications were analyzed: bleedings, wound infections, and paresis of the recurrent laryngeal nerve (RLN). Risk factors for these complications were examined by univariable and multivariable logistic regression.

**Results:**

A total of 9677 patients were included, 3871 (40%) underwent surgery to exclude malignancy and 5806 (60%) due to compression symptoms. In the multivariable analysis, the totally excised thyroid weight was an independent risk factor for bleeding. Permanent (6–12 months after the operation) RLN paresis were less common in the excluding malignancy group (*p* = 0.03).

**Conclusion:**

A range of factors interfere and contribute to bleeding, wound infections, and RLN paresis after hemithyroidectomy. In this observational study based on a Scandinavian quality register, the indication “excluding malignancy” for hemithyroidectomy is associated with less permanent RLN paresis than the indication “compression symptoms.” Thus, patients undergoing diagnostic hemithyroidectomy can be reassured that this procedure is a safe surgical procedure and does not entail an unjustified risk.

## Introduction

Nodules in the thyroid gland are common [1]. Patients may discover the nodules themselves or experience compression symptoms by them such as neck fullness, dysphagia, odynophagia, coughing, dyspnea, and voice changes as a result of the anatomy of the neck [2, 3]. A thyroid nodule may also secrete extensive levels of thyroid hormones as a solitary toxic adenoma causing hyperthyroidism [4]. Preoperative investigations include ultrasound and fine-needle aspiration where the cell formations are evaluated and classified according to scoring systems (THY, Bethesda) [5, 6]. The higher the score, the greater the risk of malignancy. Scintigraphy may also be indicated in order to determine uninodular/multinodular disease. Whether surgery is necessary or not is determined by the patients’ symptoms, the Bethesda classification, and the ultrasound examination. Patients with a thyroid nodule suspicious for malignancy are recommended hemithyroidectomy for diagnostic purposes in order to confirm or rule out a cancer diagnosis [6]. Patients in whom thyroid malignancy is not suspected but who experience compression symptoms in the throat from a predominantly unilateral thyroid enlargement are usually recommended surgery to remove half of the thyroid gland (hemithyroidectomy). Hemithyroidectomy for toxic adenomas is less common. Thus, hemithyroidectomy may be recommended for mainly two different indications, either for investigating a cancer suspicion (“excluding malignancy”) or for relieving compression symptoms.

As with any surgery, thyroidectomy carries the risk of various complications. Following hemithyroidectomy, potential major complications include infection [7, 8], bleeding [8–10], and injury to the recurrent laryngeal nerve (RLN) [8, 11, 12]. Preoperatively, the patient must therefore be informed regarding the extent of the procedure, risks, and the rationale for the surgery. Awareness of the occurrence rate and potential risk factors is therefore essential for the surgeon, both for evaluation of surgical and medical options. Studies have shown that the extent of resection (subtotal versus total) is an independent risk factor for paresis of the RLN [13, 14]. To minimize vocal cord paralysis, the surgeon’s goal is to visualize the recurrent nerve. Intraoperative nerve monitoring (IONM) has been introduced to help further detection of the RLN and reduce the risk of complication [11, 12]. With this in view, one could consider a hemithyroidectomy to be rather extensive when it is performed to exclude malignancy, since the majority (70–95%) of these patients have no symptoms and will have benign disease [15]. However, due to the lack of other definitive diagnostic options, this approach is widely used and accepted.

The risk of complications has not been studied in detail in patients undergoing diagnostic hemithyroidectomy. It could be possible that the risk for paresis RLN is particularly high in this group because surgeons might have a tendency to be as radical as possible when operating on patients with suspected cancer. We sought to investigate this hypothesis. As a control group, we chose a patient group which is also recommended hemithyroidectomy though due to compression symptoms.

## Material and methods

### Selection

In this report, we followed the STROBE guidelines [16] for observational studies.

The data were retrieved from the Scandinavian Quality Register for Thyroid, Parathyroid, and Adrenal Surgery (SQRTPA). This quality register was established in 2004 and is supported by The National Board of Health and Welfare. Across the country, 37 Swedish departments of general surgeons and ear, nose, and throat (ENT) surgeons registered more than 86% of their thyroid surgical procedures in 2019 [17]. The register contains 252 different variables divided in six different modules: demographic information of the patients, preoperative data, operative data, in-hospital postoperative data, first follow-up after 4–6 weeks, and second follow-up after 6 months. The quality of the database is evaluated by an external auditor, who visits four departments per year to validate registered data. This process has shown good data quality [18]. The study was approved by the national ethical committee (diary nr: 2019–06357) and by the steering committee of SQRTPA.

### Data

**Fig. 1:**
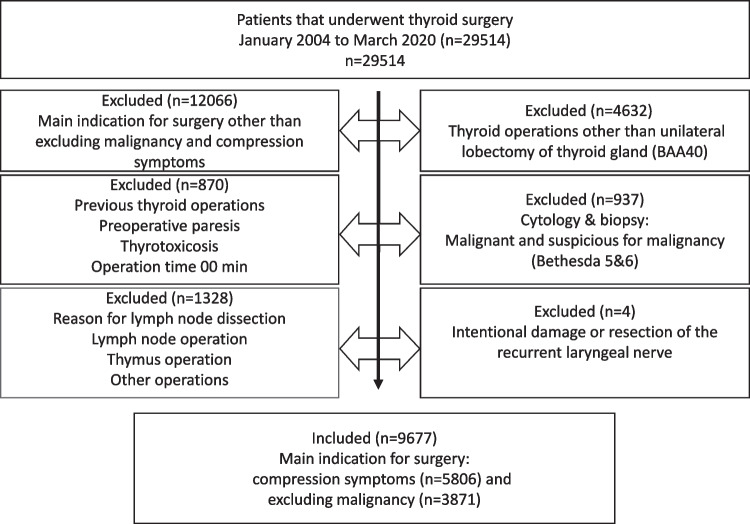
Selection of the study population

Thyroid operations were extracted from the register from January 2004 to December 2018. Twenty-nine thousand five hundred fourteen thyroid surgeries were registered. For this study, including criteria were patients who had undergone hemithyroidectomy with the International Classification of Diseases (ICD) code BAA 40 [19], either for relieving compression symptoms or for excluding malignancy. Exclusion criteria used in the selection process are shown in Fig. 1.

### Definitions

The indication “exclusion of malignancy” is defined as surgery due to a suspicious tumor of unknown dignity. Intrathoracic goiter is defined when at least 50% of thyroid length was under the jugulum. The duration of surgery was counted as skin-to-skin time. The register has recoded data on whether intraoperative nerve monitoring was used or not since 2009. Wound infection is registered when antibiotics were given or when reoperation was performed. Postoperative bleeding is registered when reoperation is performed due to a bleeding. If the patient had a normal laryngoscopy after the operation and a normal voice, no further tests were considered mandatory during the follow-up of the patient. If the patient had a RLN paresis after the operation, the patient was followed until normalization up to 6 months, and results of the laryngoscopy were registered.

The literature has shown that total thyroidectomy is associated with permanent hypoparathyroidism [20, 21]. In contrast, hemithyroidectomies almost never lead to clinical relevant hypocalcaemia [22]; therefore, this complication was not analyzed in our study.

### Statistical analysis

The not normally distributed continuous data are presented as median with interquartile range. Comparisons between groups with not normally distributed continuous data were performed by Mann–Whitney test. Comparison between the indication groups and categorical variables was analyzed using Pearson’s chi-squared test to study contingency tables. When studying contingency for nerve paresis, multiple analyses were performed on the same dependent variable, and Bonferroni correction was performed to avoid type 1 error in Table 1. Risk factors for further complications were also analyzed by univariable logistic regression. To control for confounding variables, multiple logistic regression was used to further investigate risk factors and whether they were independent or not. The following variables are known or deemed to be relevant as complications after hemithyroidectomy: the risk of postoperative infections, bleeding, and RLN paresis. They were adjusted for age, sex, duration of surgery, excised weight, intrathoracic goiter, intraoperative nerve monitoring, and main indication for surgery. The results were expressed as odds ratio (OR) with a confidence interval of 95%. All data were analyzed using SPSS® version 27 for Windows® (IBM, Armonk, NY, USA). A *p*-value of < 0.05 was considered statistically significant.

**Table 1:** Comparison of clinical variables of patients who had undergone hemithyroidectomy due to the indications “compression symptoms” and “excluding malignancy,” respectively

Variables	Total (*n* = 9677)	Compression symptoms (*n* = 5806)	Excluding malignancy (*n* = 3871)	*p*
Age* (years)	53 (42–65)	54 (42–65)	52 (41–64)	< 0.01
Sex				
Female Male	7938 (82.0%) 1739 (18%)	4849 (84.0%) 954 (16.0%)	3087 (80.0%) 784 (20.0%)	< 0.01
Duration of surgery* (min)	79 (20–456)	82 (20–456)	73 (20–330)	< 0.01
Excised thyroid gland weight* (g)	35 (18–70)	49 (27–92)	20 (12–36)	< 0.01
Intrathoracic goiter				
Yes No	877 (9.0%) 7640 (79.9%)	799 (13.7%) 4283 (73.7%)	78 (2.0%) 3357 (86.7%)	< 0.01
Wound infections				
Yes No	94 (0.9%) 9538 (98.5%)	76 (1.3%) 5730 (98.6%)	18 (0.4%) 3853 (99.6%)	< 0.01
Bleedings				
Yes	133 (1.3%)	84 (1.4%)	49 (1.2%)	
No	9508 (98.6%)	5704 (98.5%)	3804 (98.7%)	0.45
At 6–12-month follow-up Indirect or direct laryngoscopy				
Bilateral paresis	1 (0.04%)	1 (0.07%)	0 (0.0%)	
Unilateral paresis	49 (2.1%)	37 (2.8%)	11 (1.0%)	
Paresis of the left RLN	18 (0.7%)	16 (1.2%)	2 (0.1%)	0.04
Paresis of the right RLN	30 (1.3%)	21 (1.6%)	9 (0.8%)	
No RLN damage	2193 (95.7%)	1204 (94.1%)	989 (97.8%)	< 0.01
Final histology				
Benign	8416 (91.3%)	5276 (95.3%)	3140 (85.2%)	
Malignancy	801 (8.6%)	256 (4.6%)	545 (14.7%)	< 0.01

Variables are presented as the number of cases and percentages in parenthesis unless indicated otherwise

*Values are median (interquartile range)

### Missing data

Regarding postoperative bleeding, there were 36 of 9677 (0.3%) patients with missing data in this cohort. The variable wound infections had 99 (1%) patients with missing data. As these missing data are lower than 5%, multiple imputation provides insignificant benefit [23]. When investigating RLN paresis after 6–12 months, there were 38% of patients with RLN paresis from 6 weeks of follow-up which were not followed up after 6–12 months.

## Results

### Comparison of clinical variables

This retrospective analysis included 9677 patients with 5806 (60%) patients who had undergone hemithyroidectomy due to compression symptoms and 3871 (40%) patients who had undergone hemithyroidectomy due to excluding malignancy (Fig. 1). There is a predominance (82%) of the female gender, the median age was 53 years, the median duration of surgery was 79 min, the median thyroid gland weighted 35 g and was not intrathoracic (80%). The patients had an infection rate of 0.9% and a postoperative bleeding rate of 1.3%.

By comparing the two groups, there was a significant difference in age. The patients in the compression group were significantly older than the patients in the excluding malignancy group, 54 versus 52 years old (*p* < 0.01). The compression group had significantly more intrathoracic goiters (13.7% versus 2.0%) and longer durations of surgery (82 versus 73 min) (*p* < 0.01). As expected, patients in the compression symptoms group had a larger volume of the thyroid gland excised than the excluding malignancy group, 49 g versus 20 g (*p* < 0.01).

### Univariable and multivariable analysis of risk factors for postoperative infections

Wound infections occurred in 94 patients (0.9%). Older age was associated with a slightly increased risk of wound infection (Table 2). Univariate logistic analysis indicated that males had an increased risk of wound infection (OR 0.48, 95% CI 0.31–0.75).

**Table 2:** Univariable and multivariable logistic regression analysis for wound infections and bleedings

Variables	Wound infections						Bleedings					
	Univariable analysis			Multivariable analysis			Univariable analysis			Multivariable analysis		
	*p*	OR	95% CI	*p*	OR	95% CI	*p*	OR	95% CI	*p*	OR	95% CI
Age	0.005	1.02	(1.00–1.03)	0.548	1.00	(0.98–1.02)	< 0.001	1.02	(1.01–1.03)	0.244	1.01	(0.99–1.03)
Sex												
Male			1 (reference)			1 (reference)			1 (reference)			1 (reference)
Female	0.001	0.48	(0.31–0.75)	0.105	0.55	(0.27–1.13)	0.040	0.65	(0.44–0.98)	0.984	1.00	(0.40–2.49)
Duration of surgery	0.809	1.00	(0.99–1.01)	0.190	1.00	(0.99–1.01)	0.044	0.99	(0.980–0.999)	0.202	0.99	(0.99–1.01)
Excised weight (g)	0.554	1.00	(0.99–1.01)	0.570	1.00	(0.99–1.01)	0.136	1.00	(0.99–1.01)	0.014	0.99	(0.980–0.999)
Intrathoracic goiter												
No			1 (reference)			1 (reference)			1 (reference)			1 (reference)
Yes	0.011	2.03	(1.17–3.51)	0.216	1.71	(0.72–4.05)	0.029	1.74	(1.05–2.86)	0.853	0.89	(0.29–2.78)
Intraoperative nerve Monitoring												
No			1 (reference)			1 (reference)			1 (reference)			1 (reference)
Yes	0.062	1.85	(0.97–3.56)	0.183	1.74	(0.76–3.97)	0.197	0.72	(0.44–1.18)	0.453	0.74	(0.35–1.59)
Main indication for surgery												
Excluding malignancy			1 (reference)			1 (reference)			1 (reference)			1 (reference)
Compression symptoms	< 0.001	0.35	(0.21–0.59)	0.018	0.39	(0.18–0.85)	0.459	0.87	(0.61–1.24)	0.179	0.58	(0.26–1.27)

### Univariable and multivariable analysis of risk factors for postoperative bleeding

Postoperative bleeding occurred in 133 patients (1.3%). Patients who experienced postoperative bleeding were significantly older (58 versus 53 years) than the patients without bleeding (*p* = 0.001). Univariable analysis demonstrated an OR of 1.02 for age (Table 2). Male gender, duration of surgery, and intrathoracic goiter were associated with an increased risk of bleeding in the univariable analysis. The totally excised thyroid weight was an independent risk factor for bleeding in the multivariable analysis (Table 2).

### Multivariable analysis of risk factors for RLN paresis 6–12 months after the operation

There were many missing data (38%) regarding RLN paresis after 6–12 months. Therefore, we made the following adjustments: the RLN paresis after 6–12 months was analyzed with two different multivariable logistic regressions: multivariable analysis #1 with all RLN paresis that were found on the cohort without any adjustments and multivariable analysis #2 with the assumption that all missing data was counted as RLN paresis (“worst-case scenario”). The hypothesis was that if there was no difference between the two analyses then the results would be reliable.

Multivariable analyses #1 and #2 indicate that the main indication for surgery of compression symptoms is associated with higher risk of permanent RLN paresis (Table 3).

**Table 3:** Multivariable logistic regression analysis for permanent RLN paresis

Variables	Paresis at 6–12-month follow-up					
	Multivariable analysis #1			Multivariable analysis #2		
	*p*	OR	95% CI	*p*	OR	95% CI
Age	0.535	0.99	(0.99–1.00)	0.315	0.99	(0.99–1.00)
Sex						
Male			1 (reference)			1 (reference)
Female	0.249	1.16	(0.89–1.51)	0.546	1.05	(0.88–1.26)
Duration of surgery (min)	0.872	1.00	(0.99–1.00)	< 0.001	0.99	(0.99–0.99)
Excised weight (g)	0.049	1.00	(0.99–1.00)	0.938	1.00	(1.00–1.00)
Intrathoracic goiter						
No			1 (reference)			1 (reference)
Yes	< 0.001	0.45	(0.31–0.65)	0.260	1.16	(0.89–1.50)
Intraoperative nerve Monitoring						
No			1 (reference)			1 (reference)
Yes	< 0.001	2.04	(1.90–3.01)	0.577	0.95	(0.81–1.12)
Main indication for surgery						
Excluding malignancy			1 (reference)			1 (reference)
Compression symptoms	< 0.001	1.54	(1.25–1.89)	0.008	1.21	(1.05–1.40)

All the dependent variables have normal vocal cord function as reference group. Multivariable analysis #1: all RLN paresis that were found without any adjustments. Multivariable analysis #2: all missing data was counted as RLN paresis (“worst-case scenario")

### Impact on hospital stay and final histology

The complications of infection, bleeding, and RLN nerve paresis were analyzed taking into account the length of the postoperative hospital stay. The median hospital stay was 1 day in both groups regardless of the complications. The malignancy rate was analyzed in both groups, and the final histopathological examination shows a 4.6% malignancy rate for the compression group and 14.7% for the excluding malignancy group.

## Discussion

Our results show that hemithyroidectomies to exclude malignancy overall are associated with a very low risk for complications for the patients.

### Postoperative infections

Concerning both groups, wound infections occurred at a low frequency of 0.9% (94 patients). This frequency is comparable with previous findings (0.5–1.6%) [7, 8]. The reason for the increased infection rate in the excluding malignancy group is unclear. The indication excluding malignancy has a very low frequency of wound infections (18 patients, 0.4%), and this variable might jeopardize the results both on the univariable and the multivariable analysis. Longer operating times which often is associated with higher complications rates [7, 8] cannot be the reason since the operating times in the excluding malignancy group were 73 min. This time is shorter than the compression symptoms group (82 min) and the operation times of the entire cohort (79 min).

### Postoperative bleeding

Overall, postoperative bleeding occurred in 133 patients (1.3%) which matches the range of 1.0–2.1% which previous studies have reported [8–10]. Hematoma following thyroidectomies is a potentially life-threatening complication. The relation between the operation time of thyroidectomies and complications has been analyzed in a previous study where the univariate analysis showed no correlation between postoperative hematoma and the extent of surgery [24]. In our study, in agreement with that study, we did not find a statistical significance in the multivariable analysis between the duration of surgery and the frequency of bleeding.

In contrast, a higher thyroid weight was associated with a higher frequency of postoperative bleeding (*p* = 0.014) (Table 2). Patients who experienced bleeding were also significantly older. Previous studies have discussed whether older patients are more likely to be on blood hemostasis medication and thus may have an increased risk of bleeding [8]. In our study, age was not an independent risk factor for postoperative bleeding.

### Postoperative nerve paresis

After 12 months, a persisting RLN paresis is generally considered permanent, and a permanent RLN paresis might lead to voice changes, breathing difficulties, swallowing problems, reduce exercise tolerance, adjustments in lifestyle, and significantly affects the quality of life of patients [25, 26]. Only the 6–12 month follow-up regarding RLN paresis was analyzed in this study. Due to some missing data, two multivariable analyses were conducted with this variable (Table 3). Both multivariable analyses (Table 3) showed that the compression symptoms group had a higher risk of permanent RLN paresis.

Patients with a type 2 nerve injury are more likely to develop a permanent RLN paresis since it has been shown that axonal injury caused by traction of RLN which is the most common type of chronic laryngeal dysfunction has the worst prognosis [27, 28]. Unfortunately, the register does not provide information to distinguish between a type 1 (localized) RLN injury or type 2 (global) injury [29, 30].

Patients who were operated due to compression symptoms had a larger volume of thyroid gland excised as compared to patients who were operated to exclude malignancy, 49 versus 20 g (*p* < 0.01) which may appear to be obvious. Enlarged thyroid nodules are often the cause of compressive symptoms and that is why the excised gland tends to be larger. A study that was recently published by Gomez-Ramirez et al. showed that the size of the thyroid gland after total thyroidectomy did not affect the incidence of permanent RLN paresis [31]. However, the authors stated that the number of RLN injuries was very low to draw any final conclusions. On the other hand, many studies indicate that a larger size of resected thyroid specimen indeed is an independent risk factor for paresis of the RLN [13, 14, 32]. Our study strengthens these results only for a homogeneous patient group operated with hemithyroidectomy.

Postoperative RLN paresis occurred in 49 (2.1%) patients 6–12 months postoperatively (Table 1). Previous reports have shown an almost equal frequency of 2.3% for permanent RLN paresis after total thyroidectomy [33]. Bilateral paresis was noted in a few patients. The reason is unclear and not explained in the register. We can only speculate on the potential explanations such as a contralateral paresis that was not detected perioperatively. It may also be due to anesthetic complications related to the endotracheal intubation that injured one RLN while the surgeon injured the other RLN. A systematic review and meta-analysis on laryngeal injury after endotracheal intubation show that the most frequently reported mild symptom was edema (in various forms). The most frequently reported moderate injury was hematoma, and the most severe injuries were subluxation of an arytenoid cartilage and patients with vocal fold paralysis [34].

## Methodological consideration

The strength of this study was a large study sample size including 9677 patients that were operated solely with hemithyroidectomy from a Scandinavian quality register. Nearly, all the variables have been the same since the start of the register in 2004, except for IONM where the registration began in 2009. The register contains many variables with several follow-ups and is validated annually. There is a continual discussion regarding routing for input and follow-up. It should be taken into consideration that despite a small number of complication rates, we found statistically significant differences because of the large sample size, so these findings should be considered as trends rather than significance.

Unfortunately, there is no classification system such as Bethesda or THY recorded on the register. If so, then the patients could have been stratified into categories, evaluating if it was an accurate indication to perform hemithyroidectomy. If the surgeon makes an overall assessment for an unknown thyroid tumor, then the patient is selected to undergo operation for excluding malignancy. However, the histopathological examination in excluding malignancy group showed a malignancy rate of 14.7% which is equivalent to a Bethesda 3 (5–15%) or Bethesda 4 (15–30%) classification [35] being an adequate indication to diagnostic hemithyroidectomy.

Another challenge with register data is that the researcher often is not involved in the data collection. Errors may occur when entering the data. For instance, we identified a few patients where the indication for surgery was classified as “excluding malignancy” despite the fact that malignancy already had been confirmed prior surgery by fine-needle aspiration cytology. These cases were excluded from the final study population.

Surprisingly, among those patients that were diagnosed with a RLN paresis after the operation, no information was available in 38% of the nerve status at the 6–12-month follow-up. A study using the EUROCRINE register showed that 37 of 50 (74%) patients with a vocal cord paresis diagnosed earlier during follow-up did not undergo a long-term laryngoscopy [36]. Adjustments for this variable were made in our study (Table 3) and showed that the compression symptoms group had more permanent RLN paresis (6–12-month follow-up) than the excluding malignancy group.

Another methodical consideration is that our study population did not undergo systematic laryngoscopy at all the various follow-up times. Therefore, some patients who were unexamined at earlier follow-up times were later confirmed having a paresis. Studies have shown that IONM has a 98–100% [37–41] negative predictive value when comparing IONM with postoperative laryngoscopy. Therefore, the number of normal RLN in Table 3 was calculated as the number of patients with intact IONM during surgery and no clinical signs/evidence of RLN injury directly after, 6 weeks after, and 6–12 months after surgery.

## Conclusion

In conclusion, there is a range of factors that interfere and contribute to bleeding, infections, and RLN paresis after hemithyroidectomy. The frequency of these complications was low, but it was noted that patients operated due to compression symptoms were more likely to receive permanent RLN paresis than those operated to exclude malignancy. In this observational study based on a Scandinavian quality register, the indication “excluding malignancy” for hemithyroidectomy was associated with lower risk of permanent RLN paresis. Patients can however be reassured that hemithyroidectomy is a safe surgical procedure and does not entail an extensive risk.
